# Nontraumatic terminal ileal perforation

**DOI:** 10.1186/1749-7922-1-7

**Published:** 2006-03-24

**Authors:** Rauf A Wani, Fazl Q Parray, Nadeem A Bhat, Mehmood A Wani, Tasaduq H Bhat, Fowzia Farzana

**Affiliations:** 1 Department of General Surgery, Sher-i-Kashmir Institute of Medical Sciences, Srinagar, Kashmir, India; 2 Department of Accident & Emergency, Sher-i-Kashmir Institute of Medical Sciences, Srinagar, Kashmir, India; 3 Department of Social and Preventive Medicine, Govt Medical College, Srinagar, Kashmir, India

## Abstract

**Background:**

There is still confusion and controversy over the diagnosis and optimal surgical treatment of non traumatic terminal ileal perforation-a cause of obscure peritonitis.

**Methods:**

This study was a prospective study aimed at evaluating the clinical profile, etiology and optimal surgical management of patients with nontraumatic terminal ileal perforation.

**Results:**

There were 79 cases of nontraumatic terminal ileal perforation; the causes for perforation were enteric fever(62%), nonspecific inflammation(26%), obstruction(6%), tuberculosis(4%) and radiation enteritis (1%). Simple closure of the perforation (49%) and end to side ileotransverse anastomosis(42%) were the mainstay of the surgical management.

**Conclusion:**

Terminal ileal perforation should be suspected in all cases of peritonitis especially in developing countries and surgical treatment should be optimized taking various accounts like etiology, delay in surgery and operative findings into consideration to reduce the incidence of deadly complications like fecal fistula.

## Background

Perforation of the bowel especially the typhoid perforation is a serious complication and remains a significant surgical problem in developing nations. It is usually associated with high mortality and morbidity as it occurs mostly in underdeveloped countries in places where medical facilities are not readily available. Perforation of terminal ileum is a cause for obscure peritonitis, heralded by exacerbation of abdominal pain associated with tenderness, rigidity and guarding, most pronounced over right iliac fossa. However for many patients in a severe toxic state, there may be obscured clinical features with resultant delays in diagnosis and adequate surgical intervention[1].

The present study was taken to review our experience of clinical profile and management of terminal ileal perforation over past 7 years.

## Methods

The present study was a prospective study conducted by Department of General Surgery, Sher-i-Kashmir Institute of Medical Sciences, Srinagar from January 1999 to July 2005. All the patients were admitted in Accident & Emergency deptt. A thorough history was taken and detailed examination done as per proforma. Baseline investigations included hemogram, Kidney function tests, Chest & abdominal radiographs and ultrasonography. Widal test was done preoperatively only when there was a high index of suspicion of typhoid fever other wise it was done postoperatively after typical findings were noted. After thorough resuscitation, the patients were subjected to exploratory Laparotomy under General Anesthesia. Operative findings were recorded and edge biopsy at the perforation site or the resected specimen was sent for histopathological examination. The type of surgical procedure was decided on basis of operative findings. Delay in operation was the time period calculated from the time of onset of severe symptomatology like exacerbation of abdominal pain, distention and vomiting. Postoperatively the patients were followed up for any complication like fecal fistula.

## Results

There were a total of 94 cases with perforation(s) of terminal 2 feet of the ileum. 15 of these were traumatic and were excluded from the study. The mean age (± SD) of the remaining 79 patients was 34.62(±14.16) years and the male female ratio was 3:1. Pain abdomen was only constant clinical feature in all the patients (table 1). Among the investigation ultrasononography was more sensitive showing the free fluid and dilated bowel loops in 85% patients. Leucocytosis (>11 × 10^9^/L) was present in only 29.1% pts whereas only 35.4% pts had a positive Widals test. Radiology revealed pneumoperitoeum in form of gas under diaphragm in 46.8% pts.

**Table 1:** clinical features

	*Clinical feature*	No. of pts(%)
1.	Pain abdomen	79(100)
2.	Fever	45(57)
3.	Vomiting	33(42)
4.	Constipation	46(58)
5.	Dehydration	56(71)
6.	Tenderness	68(86)
7.	Distention	54(68)
8.	Rigidity	25(32)
9.	Obliteration of liver dullness	28(36)
10.	Bradycardia	08(10)
11.	Hematochezia	08(10)
12.	Palpable spleen	06(8)

Only 29% pts got operated within 24 hrs after estimated time of perforation. Mean delay in operation was 46 hours (table 2). The delay was mainly prehospital. On laparotomy only half of the patients had a single perforation in terminal ileum with majority of patients having a feculent collection in peritoneal cavity (table 3&4). Round worms (Ascariasis) were found in peritoneal cavity in 14 patients. However it is thought to be consequence of perforation and not vice versa. The final diagnosis in majority was typhoid (62%) (table 5). Those patients in whom the diagnosis could not be made and the histopathological examination revealed nonspecific inflammation were labeled as nonspecific. The surgical management of all 79 cases is depicted in table 6.

**Table 2:** perforation-operation Delay(in hrs)

	no. of patients(%)
Within 24 hrs	23(29)
24–48 hrs	27(34)
48–72 hrs	11(14)
72–96 hrs	13(17)
96–120 hrs	02(3)
20–144 hrs	03(4)

**Table 3:** number of perforations in terminal ileum

S. No	No. of perforations	no. of patients(%)
1.	One	49(62)
2.	Two	21(27)
3.	Three	03(4)
4.	>Three	06(8)

**Table 4:** peritoneal fluid collection

S. No	Peritoneal collection	no. of patients(%)
1.	No collection	06(08)
2.	Reactionary fluid	11(14)
3.	Purulent	25(32)
4.	Feculent	37(47)

**Table 5:** Etiology

S. No	etiology	no. of patients(%)
1.	Typhoid	49(62)
2.	Nonspecific	21(26)
3.	Obstruction	05(6)
4.	Tuberculosis	03 (4)
5.	Radiation enteritis	01(1)

**Table 6:** 

Operations performed	No. of pts(%)	ff	Death
Simple Closure	38(49)	2	1
Resection with end to side Ileotransverse anastomosis	33(42)	2	3
Side to side ileotransverse Anastomosis	02(3)	1	1
Resection anastomosis	05(6)	2	1
Ileostomy	01(1)	-	-

5 patients underwent re-exploration for fecal fistula. Ileostomy was done in all such cases.

## Discussion

Non traumatic terminal ileal perforation is still common as a cause for obscure peritonitis in developing and underdeveloped world although in west it is quite rare. The terminal ileal perforation presents a diagnostic dilemma to the surgeon. Laparotomy is usually carried out late often suspecting a perforated appendicitis or a duodenal ulcer.

The mean age in our study was higher than other studies[2] as the children below 12 years were excluded from the study and causes other than typhoid perforations were considered. The clinical features were similar to any other acute abdominal condition. The decision for a laparotomy was mainly clinical supplemented by investigations. However no single investigation was specific. The delay in operation since the estimated time of perforation was mainly prehospital. This is due to the fact that there most of the cases came from remote areas where the medical facilities are scarce. In cases of trauma usually there is no difficulty in management since the tissues are healthy and patients present in good clinical state Typhoid fever is predominant cause of nontraumatic perforation in developing countries. Typhoid fever, a severe febrile infectious disease caused primarly by Salmonella typhi occurs in areas where poor socioeconomic levels and unsanitary environmental conditions prevail. After ingesting contaminated food, multiplication of bacteria occurs in the reticuloendothelial system during an incubation period of 1–14 days; clinical manifestations start with bacteremia, high-grade fever, signs of systemic sepsis with characteristic normal or low blood counts and anemia^1^-the reason for low incidence of leucocytosis in our study.

Later the bacteria become localized in Peyers patches. These undergo swelling and ulceration that can progress to capillary thrombosis and subsequent necrosis. These ulcerations are always located on the antimesenteric border of the intestine and may perforate, usually in 3^rd ^week of disease. An increase in titer of agglutinins against the somatic(O) and flagellar(H) antigens of S typhi occurs (basis for Widal test). The gut in typhoid fever is edematous and friable (especially last 60 cms). There may be one or several perforations and many other impending perforations, which makes the surgery difficult[3]. Nonspecific inflammation of the terminal ileum was another predominant cause. In such cases, the operative findings were similar to that of typhoid fever but no laboratory evidence of the disease was found. The clinical picture of tuberculous perforation will be that of a diffuse peritonitis and a chest radiograph showing radiological manifestations of tuberculosis. The most common site is the terminal ileum and intraoperative differentiation from Crohn's disease is difficult. These causes are extremely rare in West where Crohn's disease, foreign bodies, perforated diverticula[4] and radiation enterits[5] are important causes. Late presentation, delay in operation(>48 hrs), multiple perforations and drainage of copious quantities of pus and fecal material from the peritoneal cavity adversely affected the incidence of fecal fistula and subsequent mortality[6,7]. The peritoneal fluid content and the delay in operation-perforation time also determine the severity of contamination and friability of gut. Various surgical procedures have been used for distal ileal perforations with variable results. Unfortunately no matter what procedure is used postoperative mortality and morbidity remains high. The most catastrophic complication being the fecal fistula and the wound dehiscence[8]. As depicted in table 5, simple debridement and closure of the perforation is most commonly employed procedure in our setup but in severely contaminated cases with friable terminal ileum (those with delayed presentation, multiple perforations, fecaloid peritonitis), obviously something more than mere closure of perforations needs to be done to reduce the incidence of most deadly complication like fecal fistula. Resection anastomosis carried a high morbidity and mortality[9]. Ileostomy would have been ideal but its maintainence in our underprivileged and the need for second operation discouraged us from its frequent use. In such circumstances end to side ileotransverse anastomosis with closure of distal stump is a better procedure[10].

## Conclusion

Terminal ileal perforation should be considered as a possibility in obscure peritonitis. In developing countries enteric perforation is a strong possibility. Early diagnosis and treatment avoids extensive procedures and is associated with lower morbidity and mortality. The preoperative diagnosis is usually made in an endemic country except in patients who are moribund; there has to be a high level of suspicion. Investigation aid in diagnosis but no single investigation is diagnostic. Non specific inflammation and tuberculosis are other causes in developing countries. The operative findings are typical with most enteric perforations on the antimesenteric border of terminal 60 cm of ileum. The operative management consists of liberal peritoneal lavage with closure of perforation. However in the patients where the terminal ileum is grossly inflamed with multiple perforations, perforation-operation delay >48 hours, fecaloid peritonitis; something more than mere closure of perforation needs to be done and end to side ileotransverse anastomosis is a better procedure.

## Competing interests

The author(s) declare that they have no competing interests.

## Authors' contributions

RW has made substantial contribution in design, acquisition and analysis of data.

FP Under his guidance the study was carried out

NB Helped in analysis and acquisition of data

MW helped in tables and management of patients

TB emergency medical specialist who enlisted most of the patients and helped in initaial management

FF studied the preventive aspects of infections in developing countries

**Figure 1:**
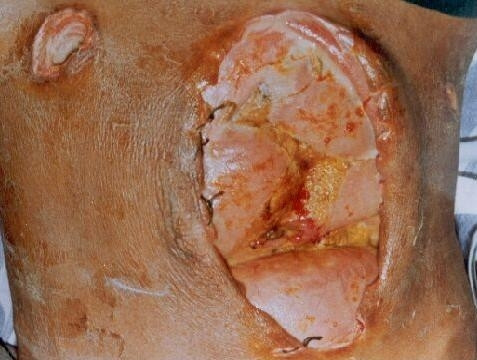
Photograph showing burst abdomen with fecal fistula in a patient of typhoid perforation following laparotomy
